# Distribution of Primary Healthcare Centers Between 2017 and 2021 Across Saudi Arabia

**DOI:** 10.7759/cureus.41932

**Published:** 2023-07-15

**Authors:** Ahmed Al-Sheddi, Shady Kamel, Abdulaziz S Almeshal, Abdullah M Assiri

**Affiliations:** 1 Field Epidemiology Training Program (FETP), Ministry of Health, Riyadh, SAU; 2 Preventive Medicine, Ministry of Health, Riyadh, SAU

**Keywords:** primary healthcare center, gini index, saudi arabia, inequalities, distribution

## Abstract

Background and aim: Access to adequate healthcare systems is seen as a fundamental human right. Therefore, healthcare services must be distributed and accessible in the most efficient way possible to those who need them the most. Primary healthcare centers are the backbone of any healthcare system as they provide essential healthcare services to the general population. Inequalities in the distribution of primary healthcare centers can lead to disparities in healthcare access and outcomes. This study aimed to assess the equity of primary healthcare centers distribution, we analyzed and evaluated the allocation of primary healthcare centers in Saudi Arabia from 2017 to 2021.

Method: This cross-sectional study utilized data from the Health Statistical Yearbook published by the Ministry of Health, Saudi Arabia, during the period of 2017-2021. The number of primary healthcare centers per 10,000 population was calculated for the 20 health regions. We used the Gini index to measure inequality in the distribution of primary healthcare centers. The Pearson coefficient was calculated to assess the correlation between the number of primary healthcare centers and the population in each health region.

Result: The overall ratio of primary healthcare centers to population decreased from 0.72 to 0.62 between 2017 and 2021. The Gini index showed relative equality in the distribution of primary healthcare centers from 2017 to 2021 with values between 0.2 and 0.3. There was a positive correlation between the population and the primary healthcare centers in Jeddah, Tabouk, and the Northern Region. However, in Riyadh, Makkah, Taif, Madinah, Qaseem, Eastern Region, Al-Ahsa, Aseer, Hail, Jazan, Najran, Al-Baha, and Al-Jouf, the correlation was found to be negative.

Conclusion: From 2017 to 2021, primary healthcare facilities are distributed fairly throughout 20 health regions of Saudi Arabia. However, there are still some disparities between provinces, and efforts must be made to ensure that primary healthcare centers are distributed equitably across the country to improve healthcare access and outcomes for all.

## Introduction

Health is a crucial aspect of daily life for all individuals, regardless of demographic factors, such as age, gender, socioeconomic status, or ethnicity. It plays a crucial role in determining one's ability to attend school or work, fulfill family responsibilities, and participate in community activities. People will often make significant sacrifices in pursuit of a longer and healthier life. Adequate access to healthcare systems is considered a basic human right. Therefore, healthcare services must be delivered to those who need them most in the most efficient manner possible [1]. The right to the highest attainable standard of physical and mental health was first recognized internationally in the 1946 Constitution of the World Health Organization (WHO), which defines health as a state of complete physical, mental, and social well-being and not just the absence of disease or infirmity. This right was further affirmed in the 1948 Universal Declaration of Human Rights and the 1966 International Covenant on Economic, Social, and Cultural Rights [2]. Primary healthcare centers are the backbone of any healthcare system as they provide essential healthcare services to the general population. Inequalities in the distribution of primary healthcare centers can lead to disparities in healthcare access and outcomes [3].

Primary healthcare centers supply the tools and services necessary for maintaining the health of citizens and improving their quality of life. The rational distribution of these centers is essential for ensuring fair distribution and optimal utilization of healthcare resources and for attaining the national aim of equalizing basic public services. Proper spatial distribution of these facilities in health regions guarantees that all demographic groups, especially the elderly and vulnerable populations, have greater access to crucial healthcare services, which leads to better overall health outcomes in urban communities and enhances the capacity to handle public health emergencies [4].

The government in Saudi Arabia made healthcare accessible by implementing a comprehensive and unified system for health-related licenses, which is closely linked to other government agencies. The regulations in the country guarantee that everyone, regardless of their condition, is entitled to top-quality healthcare without discrimination based on disability by providing universal access to healthcare through its welfare policy. The public sector offers free healthcare services to Saudi citizens with no additional financial support. The government also strives to improve the overall well-being and quality of life for individuals and their families [5]. It has a healthcare system that combines public and private services. The government's Ministry of Health is responsible for about 60% of healthcare services, which include primary care centers, hospitals, specialized services, and outpatient services [6]. To effectively increase access to primary healthcare services, it is vital to understand the distribution of primary healthcare centers in 20 Saudi health regions [7].

The Ministry of Health introduced the Ehalati system in 2017, but it only recently gained widespread use among primary healthcare providers. E-referral involves using an electronic platform to transfer patient information from a primary to a secondary or tertiary healthcare provider. This may shorten the distance between primary and tertiary care centers and improve the quality of care and efficiency of the referral process. However, the impact of this system has not been thoroughly assessed yet. A review of the literature on primary healthcare (PHC) referral services in Saudi Arabia showed that crucial information about the reasons for patient referrals is frequently under-reported. Upgrading the resources of primary healthcare centers is crucial for enhancing the quality of primary care services. To improve accessibility, implementing appointment systems, registers, and follow-up protocols are recommended [8].

Given that the National Transformation Plan 2030 aims to optimize the distribution of resources between primary healthcare centers and hospitals, it is crucial to assess primary healthcare centers and adapt them to meet the needs of the population [9]. However, research on primary healthcare capacity in Saudi Arabia is limited and often focuses on specific regions or sectors.

Similar studies from other countries have focused on the distribution of hospitals and healthcare centers aiming to assess inequality in resource distribution applying the Gini index. A Chinese study, for example, used data from the Chinese Statistical Yearbook (1999-2017) from 2008 to 2016, the Gini coefficients for healthcare institutions fluctuated between 0.150 and 0.200, fell by 0.048 (39.5%) and 0.058 (40.9%), respectively. At the same time that the number of nurses is growing at the fastest pace (109.0%), the distribution of nurses is also of the utmost importance [10]. In another study conducted in 18 states of Sudan using state-level data on health resources and health outcomes from the Sudan Health Statistical Report of 2016, the Gini coefficient values vary from 0.32 for hospitals and 0.36 for healthcare centers [11].

In Saudi Arabia, PHC has been prioritized as a core component of the newly proposed model of care, with the aim of tackling the increasing burden of non-communicable diseases. Despite this, there is a limited number of publications that review PHC capacity in Saudi Arabia, with a focus on specific regions and sectors. To address this gap, recent studies have analyzed the published PHC research conducted in Saudi Arabia quantitatively, with a focus on the distribution of research publications according to topic, time, geographical location, and institution [7]. Other studies have leveraged electronic health records to improve PHC in the country and examined patients' awareness of their rights regarding PHC [12,13]. In this context, this research aims to analyze the distribution of primary healthcare centers in Saudi Arabia from 2017 to 2021 using the Gini index and Pearson correlation, with the goal of evaluating the equity of PHC distribution and informing future policies and initiatives for enhancing PHC in the country.

## Materials and methods

Study design and setting

This study aimed to assess the distribution of healthcare centers across the 20 regions of Saudi Arabia from 2017 to 2021. The data used in the study, including population and sociodemographic information for each region, was obtained from the annual Statistical Yearbook published by the Ministry of Health [14]. The study followed a cross-sectional design. The study included all healthcare centers that fall under the regulations of the Ministry of Health in 20 regions of Saudi Arabia. The entire population, both Saudi and non-Saudi, residing in the health regions were taken into account in the analysis.

Tools and data extraction

This study used a Microsoft Excel 2016 spreadsheet to gather data from the Ministry of Health's Statistical Yearbook. The information extracted included the year, city, population, and number of primary healthcare centers. Researchers used this data to calculate the number of healthcare centers per 10,000 population in each of the 20 regions for the years 2017-2021. This study outlines the definition of healthcare centers as defined by the WHO, stating that primary healthcare centers are a comprehensive approach to health that aims to achieve the highest level of health and well-being for all, through addressing individuals' needs and providing services ranging from health promotion and disease prevention to treatment, rehabilitation, and palliative care, in a location that is easily accessible to the community. This definition is in line with the vision for primary healthcare in the 21st century, which is to work towards universal health coverage and the Sustainable Development Goals (SDGs) as defined by WHO and United Nations International Children's Emergency Fund (UNICEF) [15].

Statistical analysis

The population of 20 Saudi health regions was tracked throughout 2017-2021. The ratio of primary healthcare centers per 10,000 people was determined using the following formula: number of primary healthcare centers/10,000 population. The researchers analyzed the changes in healthcare services by determining the rate of change in the overall population and the rate of change in the total number of PHCs between 2017 and 2021.

This study aimed to assess the equity of primary healthcare centers distribution among 20 health regions. To accomplish this, the researchers employed the Gini index and Lorenz curve as analytical tools. These measures were calculated and constructed using Excel. The Gini index is a statistical variable commonly used to measure inequalities as shown in Table 1, while the Lorenz curve is a visual representation that compares disparities about a hypothetical state of "perfect" equality [11,16-18].

**Table 1:** Interpretation of Gini index values

Gini index	Evaluation
≤0.2	Perfect equality
>0.2-0.3	Relative equality
>0.3-0.4	Adequate equality
>0.4-0.5	Large equality gap
>0.5	Severe equality gap

In the context of the study, the cumulative percentage of PHC centers was illustrated on the y-axis, while the cumulative percentage of the population was illustrated on the x-axis (charts available on request). The graph also featured a diagonal straight line indicating ideal equality. The smaller the gap between the equality line and the Lorenz curve, the more equal the distribution of primary healthcare centers, the Gini index can be calculated from the graph as it represents the ratio between the area beneath the diagonal line and the curve in comparison to the entire area beneath the line of complete equality. Additionally, the researchers used Excel to calculate Pearson correlation coefficient (r) to analyze the relationship between changes in population and changes in the number of PHC centers in each region from 2017 to 2021.

## Results

In Saudi Arabia, there were 0.72 PHC centers for every 10,000 population nationwide in the year 2017; however, by 2021, that ratio had slightly declined to 0.62 PHC centers. Table 2 presents the demographic characteristics of the population of Saudi Arabia as of 2021, with a total population of 34.1 million and an annual growth rate of 0.1298%. Data from the Health Statistical Yearbook from 2017 to 2021 was analyzed, and the results are shown in (Tables 3-7). It can be seen that the trend for the number of healthcare centers per 10,000 population is decreasing over time, with fluctuations present. The total number of healthcare centers has decreased in the population, from an average of 2,361 for a population of 3,255,2336 in 2017 to an average of 2,121 for a population of 3,411,0821 in 2021 across all 20 health regions.

**Table 2:** Demographic information for the population of Saudi Arabia in 2021.

Parameter		Number
Total population		34,110,821
Saudi population		21,690,648
Male		11,028,006
Female		10,662,642
Non-Saudi population		12,420,173
Male		83,35,650
Female		4,084,523
Annual population growth rate		0.1298
Age distribution (years)	<5	7.9
	<15	24.5
	15-64	72
	>64	3.5

**Table 3:** Number of PHC centers, population, rate of PHCs per 10,000 populations, cumulative population, and cumulative PHCs of 20 Saudi health regions in 2017. Pi: percentage of primary healthcare centers; Si: cumulative population; PHC: primary healthcare

Health regions	PHCs No.	Percentage of PHCs (Pi)	Cumulative PHCS	Population	Percentage of population	Rate of PHCs per 10,000 population	Cumulative population (Si)	Si-1	(Si-1+Si)	(Si-1+Si)*Pi
Riyadh	436	18.5	18.5	8,234,302	21.0405228	0.529	21.040523	0	21.04052	389.2497
Makkah	84	3.6	22.1	8,575,578	21.9125609	0.097	42.953084	21.040523	63.99361	230.377
Jeddah	92	3.9	26	3,456,259	8.83153134	0.266	51.784615	42.953084	94.7377	369.477
Taif	121	5.1	31.1	9,87,914	2.52434596	1.22	54.308961	51.784615	106.0936	541.0772
Madinah	159	6.7	37.8	2,136,553	5.45938102	0.744	59.768342	54.308961	114.0773	764.3179
Qaseem	181	7.8	45.6	1,423,130	3.63642227	1.27	63.404764	59.768342	123.1731	960.7502
Eastern region	143	6.1	51.7	4,909,385	12.5446002	0.291	75.949365	63.404764	139.3541	850.0602
Al-Ahsa	72	3.1	54.8	1,063,112	2.71649403	0.677	78.665859	75.949365	154.6152	479.3072
Hafar Al-Batin	40	1.7	56.5	3,89,993	0.99652121	1.02	79.66238	78.665859	158.3282	269.158
Aseer	254	10.8	67.3	2,214,736	5.65915644	1.14	85.321536	79.66238	164.9839	1781.826
Bishah	85	3.6	70.9	2,05,346	0.52470594	4.13	85.846242	85.321536	171.1678	616.204
Tabouk	87	3.7	74.6	9,11,331	2.3286589	0.954	88.174901	85.846242	174.0211	643.8782
Hail	110	4.7	79.3	700,865	1.79087019	1.56	89.965771	88.174901	178.1407	837.2612
Northern region	47	2	81.3	367,558	0.93919466	1.27	90.904966	89.965771	180.8707	361.7415
Jazan	170	7.2	88.5	1,569,872	4.0113816	1.08	94.916347	90.904966	185.8213	1337.913
Najran	69	2.9	91.4	582,927	1.48951166	1.18	96.405859	94.916347	191.3222	554.8344
Al-Bahah	108	4.6	96	477,105	1.21911227	2.26	97.624971	96.405859	194.0308	892.5418
Al-Jouf	43	1.8	97.8	509,504	1.30189912	0.843	98.926871	97.624971	196.5518	353.7933
Qurayyat	17	0.7	98.5	147,550	0.37702396	1.15	99.303894	98.926871	198.2308	138.7615
Qunfudah	43	1.8	100.3	272,424	0.69610556	1.57	100	99.303894	199.3039	358.747
Total	2361	-	-	-	-	-	-	-	-	12731.28

**Table 4:** Number of PHC centers, population, rate of PHCs per 10,000 populations, cumulative population, and cumulative PHCs of 20 Saudi health regions in 2018. Pi: percentage of primary healthcare centers; Si: cumulative population; PHC: primary healthcare

Health regions	PHCs no.	Percentage of PHCs (Pi)	Cumulative PHCS	Population	Percentage of population	Rate of PHCs per 10,000 population	Cumulative population (Si)	Si-1	(Si-1+Si)	(Si-1+Si)*Pi
Riyadh	447	18.7	18.7	8,446,866	21.1207587	0.529	21.120759	0	21.12076	394.9582
Makkah	85	3.6	22.3	8,803,545	22.0126079	0.096	43.133367	21.120759	64.25413	231.3149
Jeddah	95	4	26.3	3,497,653	8.74562057	0.271	51.878987	43.133367	95.01235	380.0494
Taif	121	5.1	31.4	987,914	2.47020531	1.22	54.349193	51.878987	106.2282	541.7637
Madinah	159	6.7	38.1	2,188,138	5.47127594	0.726	59.820469	54.349193	114.1697	764.9367
Qaseem	183	7.7	45.8	1,455,693	3.63985182	1.25	63.46032	59.820469	123.2808	949.2621
Eastern region	143	6	51.8	5,028,753	12.574022	0.284	76.034342	63.46032	139.4947	836.968
Al-Ahsa	72	3	54.8	1,078,654	2.69709391	0.667	78.731436	76.034342	154.7658	464.2973
Hafar Al-Batin	40	1.7	56.5	389,993	0.97514842	1.02	79.706585	78.731436	158.438	269.3446
Aseer	254	10.6	67.1	2,261,618	5.6550072	1.12	85.361592	79.706585	165.0682	1749.723
Bishah	85	3.6	70.7	205,346	0.51345236	4.13	85.875044	85.361592	171.2366	616.4519
Tabouk	96	4	74.7	930,507	2.32666338	1.03	88.201708	85.875044	174.0768	696.307
Hail	111	4.6	79.3	716,021	1.79035713	1.55	89.992065	88.201708	178.1938	819.6914
Northern region	48	2	81.3	375,310	0.93843467	1.27	90.930499	89.992065	180.9226	361.8451
Jazan	170	7.1	88.4	1,603,659	4.00982977	1.06	94.940329	90.930499	185.8708	1319.683
Najran	68	2.8	91.2	595,705	1.48951594	1.14	96.429845	94.940329	191.3702	535.8365
Al-Bahah	108	4.5	95.7	487,108	1.21797724	2.21	97.647822	96.429845	194.0777	873.3495
Al-Jouf	43	1.8	97.5	520,737	1.30206405	0.825	98.949886	97.647822	196.5977	353.8759
Qurayyat	19	0.8	98.3	147,550	0.36893777	1.28	99.318824	98.949886	198.2687	158.615
Qunfudah	43	1.8	100.1	272,424	0.6811759	1.57	100	99.318824	199.3188	358.7739
Total	2390	-	-	-	-	-	-	-	-	12677.05

**Table 5:** Number of PHC centers, population, rate of PHCs per 10,000 populations, cumulative population, and cumulative PHCs of 20 Saudi health regions in 2019. Pi: percentage of primary healthcare centers; Si: cumulative population; PHC: primary healthcare

Health regions	PHCs No.	Percentage of PHCs (Pi)	Cumulative PHCS	Population	Percentage of population	Rate of PHCs per 10,000 population	Cumulative population (Si)	Si-1	(Si-1+Si)	(Si-1+Si)*Pi
Riyadh	415	18.4	18.4	8,660,885	21.1881144	0.479	21.188114	0	21.18811	389.8613
Makkah	80	3.5	21.9	9,033,491	22.0996631	0.088	43.287778	21.188114	64.47589	225.6656
Jeddah	95	4.2	26.1	3,567,854	8.72844966	0.266	52.016227	43.287778	95.304	400.2768
Taif	106	4.7	30.8	992,345	2.42768717	1.06	54.443914	52.016227	106.4601	500.3627
Madinah	149	6.6	37.4	2,239,923	5.47978004	0.665	59.923694	54.443914	114.3676	754.8262
Qaseem	156	6.9	44.3	1,488,285	3.64096196	1.04	63.564656	59.923694	123.4884	852.0696
Eastern region	137	6.1	50.4	5,148,598	12.5956047	0.266	76.160261	63.564656	139.7249	852.322
Al-Ahsa	70	3.1	53.5	1,082,467	2.64816293	0.646	78.808424	76.160261	154.9687	480.4029
Hafar Al-Batin	37	1.6	55.1	389,993	0.95408452	0.948	79.762508	78.808424	158.5709	253.7135
Aseer	258	11.4	66.5	2,308,329	5.64712947	1.11	85.409638	79.762508	165.1721	1882.962
Bishah	76	3.4	69.9	205,346	0.50236143	3.70	85.911999	85.409638	171.3216	582.4936
Tabouk	96	4.3	74.2	949,612	2.32314454	1.01	88.235144	85.911999	174.1471	748.8327
Hail	109	4.8	79	731,147	1.7886886	1.49	90.023833	88.235144	178.259	855.6431
Northern region	43	1.9	80.9	383,051	0.93710151	1.12	90.960934	90.023833	180.9848	343.8711
Jazan	168	7.4	88.3	1,637,361	4.00566365	1.02	94.966598	90.960934	185.9275	1375.864
Najran	69	3.1	91.4	608,467	1.48856247	1.13	96.45516	94.966598	191.4218	593.4074
Al-Bahah	94	4.2	95.6	497,068	1.21603435	1.89	97.671195	96.45516	194.1264	815.3307
Al-Jouf	41	1.8	97.4	531,952	1.30137507	0.77	98.97257	97.671195	196.6438	353.9588
Qurayyat	19	0.8	98.2	147,550	0.36096846	1.28	99.333538	98.97257	198.3061	158.6449
Qunfudah	43	1.9	100.1	272,424	0.66646202	1.57	100	99.333538	199.3335	378.7337
Total	2261	-	-	-	-	-	-	-	-	12799.24

**Table 6:** Number of PHC centers, population, rate of PHCs per 10,000 populations, cumulative population, and cumulative PHCs of 20 Saudi health regions in 2020. Pi: percentage of primary healthcare centers; Si: cumulative population; PHC: primary healthcare

Health regions	PHCs no.	Percentage of PHCs (Pi)	Cumulative PHCS	Population	Percentage of population	Rate of PHCs per 10,000 population	Cumulative population (Si)	Si-1	(Si-1+Si)	(Si-1+Si)*Pi
Riyadh	415	18.4	18.4	8,680,874	20.8695861	0.478	20.869586	0	20.86959	384.0004
Makkah	79	3.5	21.9	9,134,266	21.9595804	0.086	42.829167	20.869586	63.69875	222.9456
Jeddah	96	4.3	26.2	3,643,564	8.75944894	0.263	51.588615	42.829167	94.41778	405.9965
Taif	116	5.1	31.3	995,432	2.39310625	1.16	53.981722	51.588615	105.5703	538.4087
Madinah	156	6.9	38.2	2,343,122	5.63307177	0.665	59.614793	53.981722	113.5965	783.816
Qaseem	155	6.9	45.1	1,513,254	3.63799597	1.02	63.252789	59.614793	122.8676	847.7863
Eastern region	137	6.1	51.2	5,267,456	12.6634284	0.260	75.916218	63.252789	139.169	848.9309
Al-Ahsa	66	2.9	54.1	1,085,899	2.61059689	0.607	78.526815	75.916218	154.443	447.8848
Hafar Al-Batin	37	1.6	55.7	389,993	0.93757754	0.948	79.464392	78.526815	157.9912	252.7859
Aseer	243	10.8	66.5	2,453,226	5.89777149	0.990	85.362164	79.464392	164.8266	1780.127
Bishah	73	3.2	69.7	205,346	0.49366988	3.55	85.855834	85.362164	171.218	547.8976
Tabouk	94	4.2	73.9	959,341	2.3063403	0.979	88.162174	85.855834	174.018	730.8756
Hail	110	4.9	78.8	749,654	1.80223428	1.46	89.964408	88.162174	178.1266	872.8203
Northern region	42	1.9	80.7	396,543	0.95332432	1.05	90.917733	89.964408	180.8821	343.6761
Jazan	168	7.4	88.1	1,695,432	4.07596793	0.990	94.9937	90.917733	185.9114	1375.745
Najran	69	3.1	91.2	623,127	1.49805222	1.10	96.491753	94.9937	191.4855	593.6049
Al-Bahah	94	4.2	95.4	498,789	1.19913271	1.88	97.690885	96.491753	194.1826	815.5671
Al-Jouf	43	1.9	97.3	540,521	1.29946012	0.795	98.990346	97.690885	196.6812	373.6943
Qurayyat	20	0.9	98.2	147,550	0.3547232	1.35	99.345069	98.990346	198.3354	178.5019
Qunfudah	44	1.9	100.1	272,424	0.6549313	1.61	100	99.345069	199.3451	378.7556
Total	2257	-	-	-	-	-	-	-	-	12723.82

**Table 7:** Number of PHC centers, population, rate of PHCs per 10,000 populations, cumulative population, and cumulative PHCs of 20 Saudi health regions in 2021. Pi: percentage of primary healthcare centers; Si: cumulative population; PHC: primary healthcare

Health regions	PHCs no.	Percentage of PHCs (Pi)	Cumulative PHCS	Population	Percentage of population	Rate of PHCs per 10,000 population	Cumulative population (Si)	Si-1	(Si-1+Si)	(Si-1+Si)*Pi
Riyadh	390	18.4	18.4	8,796,548	20.964221	0.443	20.964221	0	20.96422	385.7417
Makkah	77	3.5	21.9	9,135,437	21.771873	0.084	42.736094	20.964221	63.70032	222.9511
Jeddah	92	4.3	26.2	3,685,432	8.7832422	0.249	51.519336	42.736094	94.25543	405.2983
Taif	109	5.1	31.3	997,438	2.3771269	1.09	53.896463	51.519336	105.4158	537.6206
Madinah	144	6.9	38.2	2,354,336	5.6109306	0.611	59.507393	53.896463	113.4039	782.4866
Qaseem	155	6.9	45.1	1,575,854	3.7556268	0.983	63.26302	59.507393	122.7704	847.1158
Eastern region	119	6.1	51.2	5,321,432	12.682211	0.223	75.945231	63.26302	139.2083	849.1703
Al-Ahsa	63	2.9	54.1	1,091,236	2.6006694	0.577	78.5459	75.945231	154.4911	448.0243
Hafar Al-Batin	36	1.6	55.7	389,993	0.9294441	0.923	79.475344	78.5459	158.0212	252.834
Aseer	213	10.8	66.5	2,473,226	5.8942732	0.861	85.369618	79.475344	164.845	1780.326
Bishah	64	3.2	69.7	205,346	0.4893873	3.11	85.859005	85.369618	171.2286	547.9316
Tabouk	90	4.2	73.9	973,252	2.319486	0.924	88.178491	85.859005	174.0375	730.9575
Hail	109	4.9	78.8	752,365	1.7930609	1.44	89.971552	88.178491	178.15	872.9352
Northern region	41	1.9	80.7	39,897	0.0950838	10.2	90.066636	89.971552	180.0382	342.0726
Jazan	155	7.4	88.1	1,768,732	4.2153	0.876	94.281936	90.066636	184.3486	1364.179
Najran	68	3.1	91.2	629,543	1.5003475	1.08	95.782283	94.281936	190.0642	589.1991
Al-Bahah	94	4.2	95.4	499,874	1.1913161	1.88	96.973599	95.782283	192.7559	809.5747
Al-Jouf	41	1.9	97.3	541,432	1.2903585	0.757	98.263958	96.973599	195.2376	370.9514
Qurayyat	17	0.9	98.2	147,550	0.351646	1.15	98.615604	98.263958	196.8796	177.1916
Qunfudah	44	1.9	100.1	272,424	0.6492498	1.61	99.264854	98.615604	197.8805	375.9729
Total	2121	-	-	-	-	-	-	-	-	12692.53

Throughout 2017-2021, the number of primary healthcare centers per 10,000 population in the 20 health regions has decreased generally (Tables 3-7). This decrease is particularly notable in the availability of government-run centers in both urban and rural areas. A smaller decrease was observed in Riyadh, Makkah, Tabouk, Northern region, Al-Jouf, and Eastern region, while Taif, Qaseem, Al-Ahsa, Hafar Al-Batin, Aseer, Bishah, Tabouk, Hail, Jazan, Najran, Al-Bahah, Qurayyat, and Qunfudah experienced the largest declines in number of primary healthcare centers.

The Gini indexes for the distribution of PHCs in all 20 health regions were calculated and these are summarized in Table 8. It is worth noting that the Gini index is a measure of inequality where a value of 0 represents complete equality and a value of one represents complete inequality. The Gini index of 0.27 for the 20 health regions suggests relative equality in the distribution of primary healthcare centers.

**Table 8:** Gini index for distribution of the PHCs in all 20 health regions in Saudi Arabia from 2017 to 2021. PHC: primary healthcare

Year	Gini index for distribution of PHCs in all 20 health regions
2017	0.2731
2018	0.2677
2019	0.2799
2020	0.2732
2021	0.2692
Mean	0.27262

The correlation between population and the number of PHCs was determined for various regions in Saudi Arabia from 2017 to 2021. The results revealed a weak positive correlation between population and PHCs in Jeddah, Tabouk, and the Northern region. However, in Riyadh, Makkah, Taif, Madinah, Qaseem, Eastern region, Al-Ahsa, Aseer, Hail, Jazan, Najran, Al-Baha, and Al-Jouf, the correlation was found to be negative. For Hafar Al-Batin, Bishah, Qurayyat, and Qunfudah regions, the correlation was not applicable as the number of PHCs did not change during the five years as shown in Table 9.

**Table 9:** Correlation between the number PHCs and population for the 20 health regions in Saudi Arabia from 2017 to 2021. N/A: not applicable; r: Pearson correlation; PHC: primary healthcare The Pearson correlation (r-value) is N/A when the number of PHCs did not change over the five years.

Region	r total	p-Value
Riyadh	-0.83920626	0.0756
Makkah	-0.88096233	0.0489
Jeddah	0.09633674	0.877
Taif	-0.68089329	0.206
Madinah	-0.66059378	0.225
Qaseem	-0.81297546	0.095
Eastern region	-0.80065258	0.104
Al-Ahsa	-0.80781202	0.098
Hafar Al-Batin	NA	-
Aseer	-0.79037918	0.111
Bishah	NA	-
Tabouk	0.2625288	0.670
Hail	-0.50958717	0.381
Northern region	0.52319749	0.365
Jazan	-0.87765636	0.050
Najran	-0.22216716	0.719
Al-Bahah	-0.92608987	0.023
Al-Jouf	-0.52621556	0.362
Qurayyat	NA	-
Qunfudah	NA	-

Additionally, Table 9 shows that the number of PHCs in the Jeddah, Tabouk, and Northern regions increased as the population increased, but in the Riyadh, Makkah, Taif, Madinah, Qaseem, Eastern region, Al-Ahsa, Aseer, Hail, Jazan, Najran, Al-Baha, and Al-Jouf regions, the number of PHCs decreased as the population increased. This indicates that there may be a need to re-evaluate the distribution of PHCs in these regions to ensure that the population has adequate access to healthcare services. Overall, the data suggests that there is a complex relationship between population and the number of PHCs in Saudi Arabia, and further research may be needed to fully understand and address this issue.

## Discussion

Primary healthcare centers serve as the starting point for patients in accessing healthcare services. Many countries measure the performance of their primary healthcare system using health indicators. WHO emphasizes the importance of primary healthcare, viewing it as a crucial factor in promoting overall health and a vital component of an effective healthcare system. Regardless of resource availability, countries that prioritize primary healthcare have made significant progress toward the SDGs [7]. This study aimed to investigate the current state of PHCs distribution among 20 regions in Saudi Arabia from 2017 to 2021 and to evaluate the fairness of PHCs distribution with population growth. Results revealed a general decline in the number of PHCs in both urban and rural regions. However, the decline in the ratio of PHCs per 10,000 people was more gradual and varied over time, indicating that there has been a slight reduction in the ratio of PHCs to population over time.

Additionally, in this study, we found disparities in the distribution of PHCs across different regions, with some regions having a higher or lower number of PHCs per population than others. Furthermore, the study observed no correlation between population growth and PHC distribution, areas with higher population growth having a greater need for PHCs to ensure adequate healthcare services. Overall, the study highlights the need for further investigation and efforts to ensure that PHC distribution is fair and sufficient to meet the healthcare needs of the population. Such studies should be conducted on a regular basis to assess progress in the equality of distribution of health facilities.

The evidence from this study suggests that health authorities are able to reach residents in Saudi Arabia despite the geographic spread of health services. Similar results were seen in Saudi Arabia in a prior research by El-Farouk to assess the geographic distributional inequality of health centers, hospitals, hospital beds, and physicians between 1997 and 2013, the average Gini index for PHCs was 0.224 for the time period [19]. This show that Saudi Arabia's healthcare policymakers have been able to maintain a roughly equal distribution of PHC facilities throughout all the regions despite the country's expansion.

The study identified that among the 20 health regions in Saudi Arabia, Jeddah, Tabouk, and the Northern region have low increases in the ratio of PHCs to population. However, it is worth noting that these regions have a smaller increase in population compared to other regions. On the other hand, some regions with larger populations have a fluctuating pattern in the ratio of PHCs to the population due to disproportionate increases in both the number of PHCs and population size. The regional disparities in the population-to-center ratio suggest that certain areas of the country may be underserved in terms of primary healthcare services. Policymakers and healthcare providers should prioritize these areas and invest in the establishment of new primary healthcare centers to address the unmet needs of the population.

The ideal ratio of PHCs to population depends on several factors, such as the healthcare needs of the population, the geographic distribution of the population, and the availability of healthcare resources. There is no universal standard for the ideal ratio of primary healthcare centers to population, as this can vary depending on the context. However, PHCs should be easily accessible to the population they serve to ensure that essential health services are available to all. Ultimately, the ideal ratio of primary healthcare centers to population should be determined based on a comprehensive assessment of the healthcare needs and resources of the population being served [20]. The quality of healthcare services provided by PHCs is not solely determined by the number of centers available. Other factors such as the availability of healthcare resources, the training and qualifications of healthcare providers, and the overall healthcare system infrastructure can also influence the quality of care provided. In the current era of PHCs providing only access to healthcare is not enough, the goal is to provide quality care that is safe, effective, people-centered, timely, efficient, equitable, and integrated [21].

It is important to understand that this analysis is limited to primary healthcare centers and does not take into account other forms of healthcare, such as hospitals or specialty clinics. A more comprehensive analysis of healthcare accessibility would take into account all forms of healthcare, not just primary healthcare centers. Additionally, this analysis is limited to the years 2017-2021 and the distribution of primary healthcare centers may change over time. It is also important to consider other factors that may affect the accessibility of primary healthcare centers, such as transportation and socioeconomic status. Overall, the Gini index provides valuable insights into the equity of PHCs distribution across Saudi Arabia, but it is important to consider other factors and conduct a more comprehensive analysis to fully understand the accessibility of healthcare in a given region. Using secondary data for the study may have limitations; however, the data used for the study was provided by government institutions. It is still valuable in providing insight into the issue of distribution and accessibility of PHCs to inform policy decisions and guide future research in this field.

## Conclusions

From 2017 to 2021, primary healthcare centers are distributed fairly throughout 20 health regions of Saudi Arabia. The Gini index showed relative equality in the distribution of primary healthcare centers from 2017 to 2021 with values between 0.2 and 0.3. However, there are still some disparities between provinces and efforts must be made to ensure that PHC centers are distributed equitably across the country to improve healthcare access and outcomes for all.

It is important to note that the quality of healthcare services provided by PHCs is not solely determined by the number of centers available. Other factors such as the availability of healthcare resources, the training and qualifications of healthcare providers, and the overall healthcare system infrastructure can also influence the quality of care provided.
